# Developing an integrated school eye health programme in Pakistan

**Published:** 2017

**Authors:** Sumrana Yasmin, Khalid Saifullah, Hassan Minto

**Affiliations:** Regional Director, South East Asia & Eastern Mediterranian Public Health, Brien Holden Vision Institute, Pakistan; Program Manager, Brien Holden Vision Institute, Pakistan; Director of Programmes: Child Eye Health & Low Vision, Brien Holden Vision Institute

**Figure:**
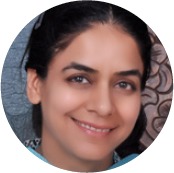
Sumrana Yasmin

**Figure:**
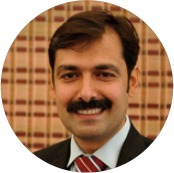
Khalid Saifullah

**Figure:**
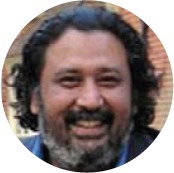
Hassan Minto

**Given the paucity of human resources for eye health, especially in developing countries, innovative approaches need to be developed and primary eye care components strengthened.**

**Figure:**
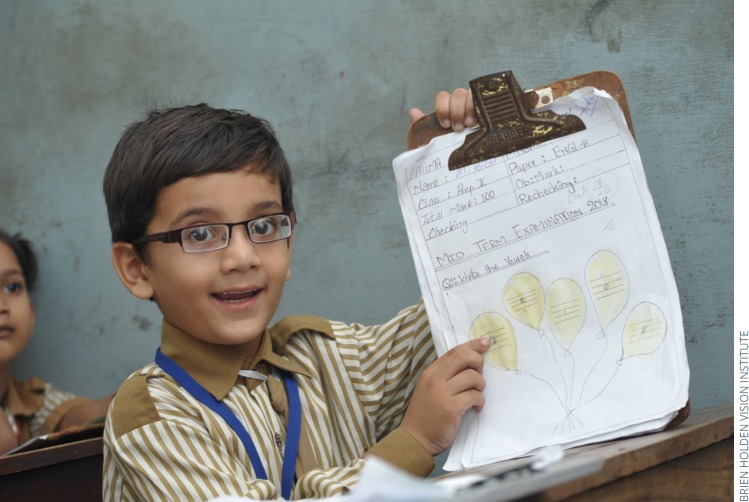
A child after vision correction as part of a school eye health programme. PAKISTAN

The importance of a child's eye health cannot be understated. Poor vision can affect a child's educational attainment and thereby have a negative impact on a child's future life.^1^ As data from the Sustainable Development Goals suggests, educational attainment has a direct impact on future indicators for individual (and national) economic growth, and, more importantly the health and educational outcomes of children.^2^ Eye health is an essential part of a school health programme. It should be comprehensive and respond to a wide range of eye conditions and diseases prevalent in the project area. Given the paucity of human resources for eye health, especially in developing countries, innovative approaches need to be developed and primary eye care components strengthened. This could include training non-clinical personnel like school teachers and community-based workers to undertake basic eye health screening and appropriate referrals to the primary eye health system for further management.

This paper describes the institutional approach to a school eye health programme in Pakistan. The programme engages multiple stakeholders to provide primary eye care and this has led to improved access, especially for children in rural communities.

The estimated population of Pakistan is 195 million^3^ − 41% of which are below the age of 18 years^4^ and among them 90% are enrolled at schools.^5^ More than half of this population lives in rural areas. Pakistan is ranked 147^th^ on the human development index.^6^ More than one-third of the population lives below the national poverty line. According to the recently conducted Rapid Assessment of Refractive Error in Children in Pakistan (2016–2017) by the Brien Holden Vision Institute, the prevalence of significant refractive error is 5.4% in the age of 5–15 years (in a study submitted for publication).

However, no reliable data exists on the prevalence of ocular morbidity in children in Pakistan and it is estimated that more than 10% of the children suffer from some form of ocular morbidity, predominantly due to Conjunctivitis, Trachoma, and Ocular Trauma. In countries like Pakistan, eye care is normally considered a subject of secondary and tertiary health interventions; increasing the cost to patients and the health system. The secondary and tertiary eye health facilities are inadequate and inequitably distributed across the country. The need for the development of primary eye health services is crucial. It requires capacity building of diverse cadres in public and private sectors to strengthen the primary eye health workforce. This needs inter- and intra- departmental partnerships with the public and private sectors, and civil society.

School health and school eye health is neither a prioritised theme for public sector education and health departments nor is eye health integrated in the school health agenda. Only Non-Governmental Organisations (NGOs) are implementing the school eye health (SEH) programmes in collaboration with public sector health and education departments in select geographical locations. In most cases, NGOs work with the education department while implementing their SEH programmes. In Pakistan, a number of other public and private sector organisations that run different types of schools, are normally discounted from such SEH programmes. In order to develop a comprehensive SEH programme that can claim to be inclusive, all such actors need to be involved that are directly or indirectly engaged in education and health interventions. There is a dire need to develop a comprehensive and practical framework that can cater to diverse needs and engage various stakeholders in education and health sectors to deliver integrated school eye health programmes.

## School Eye Health Programme

School eye health is an effective strategy for implementing eye care programmes including correction of vision impairment due to uncorrected refractive error. Early intervention can prevent the child from losing vision due to Amblyopia. Given the huge unmet need and lack of standardised approaches to school health in Pakistan, the Brien Holden Vision Institute (the Institute) prioritised SEH as a key focus for its child eye health intervention. The SEH strategy emphasises the following:

- To ensure accessibility of high quality eye health services to all children.
- To actively advocate the importance and integration of school eye health initiatives into existing education and health systems.
- To strengthen the institutional capacity of key stakeholders in planning, implementation, and effective delivery of SEH interventions leading to policy development.

The programme demonstrates the potential for adoption by the government and other eye care and non-eye care development partners.

## Roles of various stakeholders

Developing a comprehensive school health programme requires partnerships both at the substructure and superstructure levels. Partnership at the level of superstructure results in ownership of the programme, its sustainability and integration of best practices into the policy discourse. Active engagement at substructure level ensures the effective transfer of knowledge and skills to clinical and non-clinical primary cadres, extensive school screening, community participation, and appropriate referrals. In the implementation of a SEH programme, diverse organisations can be engaged to reach out to a larger number of children. The table below explains the types and roles of the different organisations engaged in the programme to promote SEH (Table 1).

A comprehensive SEH programme, developed with the active ownership of diverse stakeholders serves the following key functions:

- Links schools, communities, government departments, private sector and civil society organisations.
- Increases early detection of vision impairment among children and facilitates provision of appropriate solutions.
- Raises eye health awareness at school, community and organisation levels.
- Enables children with vision impairment to continue their education.
- Promotes inclusiveness by providing primary eye care to all children regardless of any difference.

**Table 1:** Type and role of the different organisations engaged in the programme to promote SEH

Nature of the organisations	Role in school eye health
**Public sector** Ministries of Education, Health, Social Welfare, Child Welfare District Governments School management committees Police departments	Integrating eye health into school health and primary health systems Resource allocation for spectacle provision for those children who cannot afford them Introducing SEH in schools run by government departments Permitting different cadres for trainings on SEH to provide primary eye care at department, community and school levels Generating evidence to further incorporate in policy discourse
**Civil society organisations** Local non-governmental organisations (NGOs) International non-governmental organisations (INGOs) working on education, health, hygiene, gender, livelihoods and child rights	Development of demonstration approaches based on best practice Integrating child eye health into community-based projects on education, health, hygiene, sanitation, child rights, and other relevant issues Increasing eye health awareness among communities and schools in the project area Promoting SEH in schools run by NGOs and associated communities Advocating and lobbying at local levels with education and health departments
**Private sector** Social entrepreneurs Private eye hospitals and businesses	Integrating SEH in regular eye and health services School screening and referring children with eye care needs to secondary and tertiary eye health facilities Ensuring the availability of affordable and good quality spectacles frames, lenses and accessories for children of all ages
**Media (print, electronic, social media)**	Disseminating information on SEH to broad audiences Promoting targeted eye health education and health promotion Mobilising the communities in rural areas
**Academia, particularly, health professionals** **Eye health professional forums**	Integrating SEH in eye health curriculums at all levels Facilitating the engagement of optometry and ophthalmology graduates/undergraduates in SEH programmes especially in trainings and outreach activities

**Figure:**
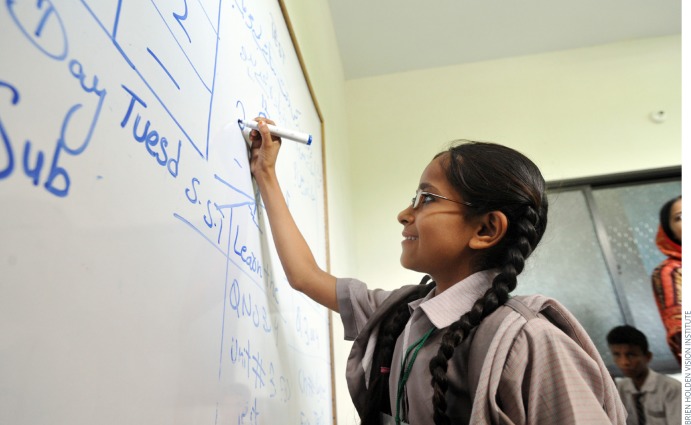
Provision of good quality eye care enhances the confidence of children, especially girls. PAKISTAN

In the last three years, through its SEH programme, the Institute has reached 520,000 children including 47% girls by engaging with public and private sector organisations, academia and civil society organisations. The Institute has dispensed over 25,000 spectacles and 1,300 low vision devices to boys and girls in the programme's geographical areas. Contributing to the broader SEH agenda along with other civil society organisations, the Institute has built the capacities of more than 2,500 diverse cadres including teachers, social workers, NGO employees, hygiene promoters and others in child eye health and vision screening.

## Key Lessons

A SEH system needs to be compatible with local culture and policy environment. Factors which facilitated the effective implementation of SEH programmes in Pakistan are:

- Partnership with education and health departments to build synergies; bring ownership, ease of getting necessary approvals, continuity and sustainability.
- Training teachers in primary eye health and appropriate referrals to build a community-based cadre, given the scarcity of human resources for health care.
- Availability of training material in the local language enables non-clinical cadres to learn and understand SEH better.
- Better-equipped trainers will keep the diversity of the participants in mind and provide local and contextual examples.
- Identifying potential women and men, especially in rural areas, to establish micro-social enterprises, contributes to reaching more children both in and out of schools.
- Strict implementation of a code of conduct for child protection reduces the risk of harm to children and presents the staff engaged in SEH as positive role models for children.
- Eye health, being a high priority subject, is acceptable to all stakeholders involved in the community development process.
- Eye health is thematically and operationally compatible with regular education, health and hygiene programmes.
- Media is a valuable partner in eye health awareness especially in rural communities.
- In low-income countries, different departments in public and non-government sectors run their own schools. These schools need to be engaged in broader school health programmes.

## Challenges

There were several challenges in implementing this programme and the following lessons were learnt:

- An enabling policy framework to support education and health sector reforms is needed in Pakistan.
- Education and health departments work independently, with little coordination between them. An effective SEH programme requires efficient coordination among key stakeholders.
- Non-availability of data related to schools run by NGOs, social welfare and other departments.
- A mechanism to provide financial assistance to children whose families cannot afford to pay for services including spectacles.
- Developing consensus to implement a broad monitoring, evaluation and learning framework requires considerable discussion.

**Figure 1:**
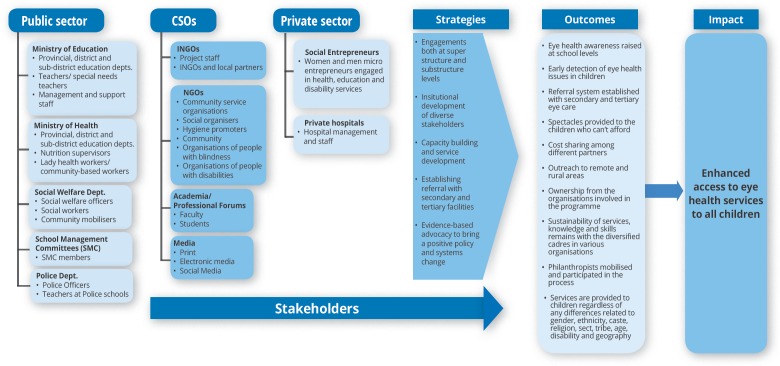
School eye health system with non-clinical and diverse stakeholders

## Conclusion

In order to be effective and sustainable, a SEH programme must be integrated within the education and health systems, particularly within the school health programme. It is crucial to engage with diverse stakeholders for the implementation of SEH in low income countries, especially ones which have large populations and a large number of school-going children. Developing countries also have fewer eye health workers, thus creating a space and need for other cadres to be involved in eye care. Coordination is essential at all levels. Roles and responsibilities need to be clearly defined for this system to function effectively. Integrating SEH into the broader health agenda will also help in the pursuit of Sustainable Development Goals.
